# siRNA suppression of hTERT using activatable cell-penetrating peptides in hepatoma cells

**DOI:** 10.1042/BSR20140145

**Published:** 2015-03-18

**Authors:** Hua Li, Jiwen He, Huimin Yi, Guoan Xiang, Kaiyun Chen, Binsheng Fu, Yang Yang, Guihua Chen

**Affiliations:** *Department of Hepatic Surgery, the Third Affiliated hospital, Sun Yat-san University, Guangzhou 510630, China; †Department of General Surgery, Guangdong No. 2 Provincial People's Hospital, Guangzhou 510317, China

**Keywords:** cell-penetrating peptides (CPPs), liver cancer, small interfering (siRNA), tumour targeting, aCPPs, activatable cell-penetrating peptides, CPPs, cell-penetrating peptides, DMEM, Dulbecco's modified Eagle medium, FBS, Fetal Bovine Serum, GAPDH, glycerladehyde 3-phosphate dehydrogenase, GFP, Green fluorescent protein, HPLC, High Performance Liquid Chromatography, hTERT, human telomerase reverse transcriptase, MMPs, matrix metalloproteinases, MPP2, matrix metalloproteinase-2, PBS, Phosphate Buffer Solution, RT, reverse-transcription, siRNA, small interfering RNA

## Abstract

Activatable cell-penetrating peptides (aCPPs) allow non-viral, low cytotoxic and selective delivery of compounds into target cells for cancer therapy. In tumour cells, up-regulation of human telomerase reverse transcriptase (hTERT) frequently occurs and is being considered as a target in cancer diagnosis and treatment. siRNA sequence that target hTERT mRNA can silence the gene and reduce hTERT protein expression to reduce cell proliferation and inhibit cell growth. In our study, we tested a matrix metalloproteinase-2 (MPP2) aCPP in delivering hTERT siRNA into hepatocellular carcinoma cells (SMMC-7721) to silence the hTERT gene. Cultured SMMC-7721 cells were transfected with a complex of aCPPs and hTERT-specific siRNA-encoding or control plasmids. Compared with cells treated with the complex of control plasmid–CPPs, cells treated with the hTERT-specific siRNA-encoding plasmid–CPP complex had a prolonged G_1_-phase, but a shorter G_2_/S-phase, indicating a G_1_-arrest. Treatment with the hTERT-specific siRNA resulted in a significant decrease (by 26%; *P*<0.05) in hTERT mRNA levels. The aCPPs tested in this study provides a non-viral delivery of siRNA into cancer cells to silence target genes in cancer therapy.

## Abstract 2

In the present study, we delivered human telomerase reverse transcriptase (hTERT) siRNA into SMMC-7721 hepatoma cells using a matrix metalloproteinase-2 (MMP2)-activatable cell-penetrating peptide (aCPP). The siRNA subsequently induced down-regulation of the hTERT gene and G_1_-arrest, implicating the utility of this delivery system in cancer therapy.

## INTRODUCTION

Cell-penetrating peptides (CPPs) are short peptides, typically 5–30 amino acids in length that can carry a variety of substances into the eukaryotic cytoplasm and even into the nucleus, although having little cytotoxicity [1]. Because of this, CPPs have been explored widely for their potential in various therapeutics [2]. In particular, they have been applied and are being developed for application in *in vivo* and *in vitro* experiments involving malignancies such as breast cancer, ovarian cancer, prostate cancer and colon cancer [3–6].

CPPs are capable of delivering a variety of molecular cargo across the cell membrane for cancer therapy [7]. The primary limitation encountered is the lack of cell-type specificity, which leads to undesired systemic side effects [8]. To selectively target cancer cells, CPPs have been adapted for cell-specific delivery of associated cargos with the use of homing peptides, antibody derivatives or targeting peptides that recognize receptors or intracellular enzymes specifically expressed/overexpressed in cancer cells [5]. The concept of targeted activation of CPPs was pioneered by Jiang et al. [5], in which they constructed aCPPs (aCPPs) that can only be activated when the linkers are cleaved by matrix metalloproteinases (MMPs), releasing the CPPs from inhibitory polyanions. Following cleavage, the activated CPPs and cargo are able to attach and cross cell membranes. This strategy provides a solution to selectively target tumours in cancer therapy, as MMPs are the most well-known proteases overexpressed by tumours. A majority of malignant tumours express MMP2; the expression of which is associated with the degree of malignancy, tumour angiogenesis, invasiveness and metastasis [9–14].

Telomerase activity is aberrantly high in over 90% of human cancers. The expression of human telomerase reverse transcriptase (hTERT), the catalytic telomerase subunit, is a key determinant of telomerase activity in cancer cells [15,16]. Aberrant transcriptional up-regulation of hTERT expression is thought to account for enhanced telomerase activity in cancer, including hepatocellular carcinoma that may contribute to cellular immortalization and carcinogenesis [17,18]. Therefore, hTERT expression is a promising target for anti-cancer therapies such as RNA interference [15]. Specific inhibition of telomerase expression in cancer cells can reduce telomerase activity and ultimately inhibit cell growth and induce apoptosis in the targeted cancer cells.

siRNA are short RNA molecules that can target mRNAs for degradation, making them ideal for development as therapeutic agents to correct aberrations in gene expression in cancer [19]. Many delivery systems for siRNA-based therapies have been devised or are currently under development, including both synthetic and natural (bacterial or viral) carriers [19,20]. CPPs are one of the non-viral siRNA carriers and as an activatable construct, can be used to deliver siRNA or siRNA-encoding plasmid DNA cargo to the targeted tumour tissue [21].

In the present study, we used a MPP2 aCPP to deliver hTERT siRNA into SMMC-7721 hepatoma cells, known to overexpress MMP2 [22], to silence the hTERT gene. We then assessed the penetration efficiency and cellular effects of the CPPs delivery system on hTERT expression and SMMC-7721 cell growth.

## MATERIALS AND METHODS

### Synthesis of CPPs and fluorophore labelling

The polypeptide sequence was synthesized as described in previous reports [5,23]. Breifly, peptides were synthesized on a peptide synthesizer (Pioneer Peptide Synthesis System, Applied Biosystems) according to the manufacture's standard protocols for Fmoc solid-phase synthesis. For fluorophore labelling, the synthesized peptides were mixed with dimethylformamide-(Samsung Fine Chemicals Co. Ltd.) dissolved 5-carboxy-tetramethylrhodamine (Sigma), 1-hydroxy-benzotriazole and diisopropyl carbodiimide (GL Biochem) in a molar ratio of 3:3:4 for 60 min followed by washed with washing solution [95% trichloroacetic acid, 2.5% 1,2-dimercapto ethane (GL Biochem), 2.5% de-ionized water (v/v)]. The molecular mass of the peptide product was analysed by Agilent 6410 triple quadrupole mass spectrometer (Agilent). The product was purified by a Prominence LC-20A HPLC system (Shimadzu) using a C18-column dried by a vacuum dryer (VOS-30A, STIK GROUP LLC) for 24 h and stored at −20°C for cryopreservation. The sequence of the targeted CPP synthesized in the present study was N-terminal-[EEEEEEE-GALGLP-RRRRRRRRKKR]-C terminal. The connecting peptide sequence was PLGLAG, which is an MMP-2-specific proteolytic recognition fragment and has a hydrolytic cleavage point between G and L.

### Cell culture

SMMC-7721 hepatoma cells (A.T.C.C.) were cultured in high-glucose Dulbecco's modified Eagle medium (DMEM, Gibco) containing 10% FBS (Hyclone), 100 units/ml penicillin and 100 μg/ml streptomycin (Gibco) in incubator at 37°C in an atmosphere of 5% CO_2_.

### Recombination of plasmid vector

The pRNAT-U6.1/Neo plasmid (preserved by The Liver disease Laboratory of Sun Yat-sen University, Third Affiliated Hospital), which also encodes a coral GFP marker for monitoring transfection efficiency, was linearized by digestion with DNA endonuclease (Sigma). Then, T4 DNA ligase (Sigma) was used to connect the DNA template encoding the hTERT-specific siRNA transcript to the linearized pRNAT-U6.1/Neo plasmid to generate the siRNA-expressing plasmid.

### Preparation of CPP–siRNA plasmid complexes

The siRNA-encoding plasmid and penetrating peptides were diluted in HEPES buffer (10 mmol/l HEPES, 150 mmol/l NaCl, pH 7.4) at different molar ratios of CPP to plasmid DNA (1:1 to 30:1) and blended gently. After incubating the mixture at room temperature for 30 min, the formation of the plasmid–CPP complex was monitored by resolving the complexes using gel electrophoresis on 1% agarose gels (Sigma). The shifted plasmid bands were visualized by Ethidium Bromide staining and analysed using the Labworks gel imaging system (GDS800, UVP). The agarose gel shift assay was used to evaluate the molar ratio of the plasmid DNA and the CPP that yielded the maximum shift in mobility.

### Transfection of cells with siRNA-encoding plasmid–CPP complexes

Cells were divided into the experimental and control groups. A total of 2 μg of plasmid was used to transfect into SMMC-772 cells in a well of six-well culture plate. In the experimental group, cells were transfected with the siRNA plasmid–CPP complexes, whereas in the control group, the cells were transfected with the control plasmid (vector lacking the siRNA expression cassette)–CPP complexes. The day before transfection, SMMC-7721 cells were seeded on to six-well culture plates at a density of 5×10^5^ cells/well in high-glucose DMEM and incubated at 37°C in an atmosphere of 5% CO_2_. When the cells reached 80%–90% confluence, the cells were washed twice with 1 ml of antibiotic-free DMEM containing FBS before transfection with the plasmid–CPP complexes. The cells were incubated for 3 h before 1.5 ml of antibiotic-free high-glucose DMEM containing FBS was added. The GFP fluorescence in the cells was observed using a fluorescent microscope (TE2000-U, Nikon) and images of the cells were captured using a camera (Canon) attached to the microscope 24, 48 and 72 h after transfection.

### Flow cytometry

The cells were collected 48 h after C11 and C12 transfection. The cells were then collected using centrifugation, the supernatant was removed and the cells were rinsed twice with pre-cooled PBS. The cells were fixed by adding pre-cooled 70% ethanol and incubating overnight at 4°C. The cells were collected by centrifugation and rinsed twice with PBS. A cell suspension in PBS was prepared and the cell concentration was adjusted to 1×10^6^ cells/ml. The cell suspension was treated with RNase and stained with 1.5 ml of propidium iodide (PI; 5 mg/ml, Sigma) solution for 1 h. After filtration, the cells (1×10^4^ cells per sample) were analysed using flow cytometry with the BD FACSCalibur system (BD).

### Reverse-transcription PCR

Single cell suspensions of the transfected cells were prepared 48 h after transfection by digesting with 0.25% trypsin (Sigma). The cells were centrifuged at 200g to remove the supernatant, rinsed with PBS and the centrifugation and wash procedure was repeated. RNA was extracted from cells using Trizol solution (1 ml/cell pellet; Invitrogen) using the manufacturer's instructions. The RNA (1 μg/sample) was reverse-transcribed to obtain the cDNA using the PrimeScript™ RT Reagent Kit. Then, the expression of hTERT mRNA was analysed by real-time PCR using 1 μl of cDNA in a semi-quantitative PCR instrument (LightCycler 480; Roche) with the SYBR Green I detection method (Invitrogen; PCR details are listed in Table 1). The following primers were used for the real-time PCR: 5′-GCGTTTGGTGGATGATTTCT-3′ and 5′-CAGGGCCTCGTCTTCTACAG-3′ (hTERT); 5′-ACCACA-GTCCATGCCATCAC-3′ and 5′-TCCACCACCCTGTTG-CTGTA-3′ (GAPDH). The relative hTERT mRNA expression level in the experimental group was calculated using the ΔCt method (where, ΔCt=Ct [hTERT] − Ct [GAPDH]; ΔΔCt=ΔCt [experimental group] − ΔCt [control group]; and relative expression level of hTERT mRNA in the experimental group=2 − ΔΔCt). The expression levels of other mRNAs were normalized to those of glycerladehyde 3-phosphate dehydrogenase (*GAPDH*) mRNA, the internal control. The formula for calculating the inhibition rate of hTERT mRNA: Inhibition rate (%)=(1 − [Relative expression of hTERT mRNA in the experimental group/control group]) × 100%. All primers were synthesized by Invitrogen Biotechnology Co. Ltd.

**Table 1:** Real time-PCR reaction mixture

Reagents (concentration)	amount (μL)
Premix Ex	
Taq^TM ^(2×)	10.0
Upstream primer (10 μM)	0.4
Downstream primer (10 μM)	0.4
cDNA template	1.0
RNase Freed H_2_O	8.2
Total	20

### Analysis of cell proliferation

The cells were seeded in 96-well culture plates at a density of 5000 cells/well and cultured overnight. The cells were transfected with the plasmid–CPP or control plasmid–CPP complexes for 48 h. Then, 20 μl MTT solution (5 mg/ml; Applygen Technologies Inc.) was added to each well and the cells were incubated for 4 h. After aspirating the supernatant carefully, 100 μl MTT solvent was added and the plates were incubated again for 4 h. The complete dissolution of the purple crystal deposits of formazan was confirmed by observation under a light microscope and the absorbance of the samples at 490 nm was measured using recorded microplate reader (ELx800; BioTek).

### Statistical analysis

All data are represented as mean±S.D. Statistical analysis was performed using the SPSS software package version 13.0 (SPSS USA). Statistical analysis of multi-sample means was performed with one-way ANOVA. Pair-wise comparison of independent sample means was performed with the group *t*test. Values of *P*<0.05 were considered statistically significant.

## RESULTS

### Preparation of siRNA-encoding plasmid–CPP complexes

**Figure 1:**
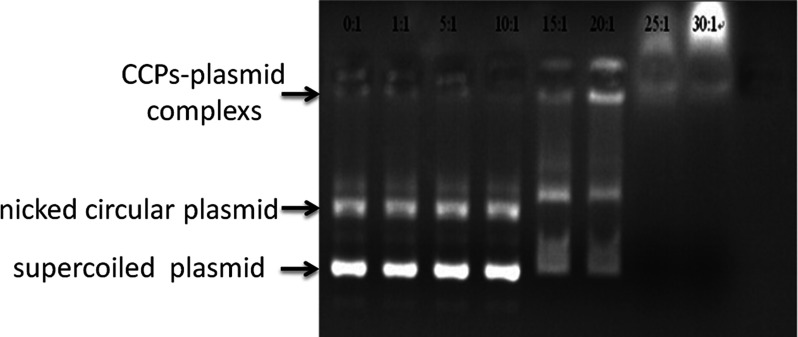
EMSA Complexes of the MMP-2 aCPP and hTERT-specific siRNA-encoding plasmid DNA were prepared at molar ratios of 1:1, 5:1, 10:1, 15:1, 20:1, 25:1 and 30:1. The formation of a stable complex was evaluated by monitoring the shift in electrophoretic mobility on 1% agarose gels, followed by visualization and analysis with Ethidium Bromide staining, UV transillumination and the Labworks software package. The maximum shift was obtained at a molar ratio of 30:1; therefore, this molar ratio of MMP-2 aCPP and hTERT-specific siRNA-encoding plasmid DNA was used for subsequent analyses.

In gel electrophoresis, small nucleic acid fragments migrate toward the anode more rapidly than those that have formed complexes with proteins. Gel shift assay, followed by visualization of DNA with autoradiography, was used to evaluate the optimal molar ratio of the mixture of the siRNA-encoding plasmid and CPP for forming the most stable complex. The data showed that the optimum molar ratio of the CPP–siRNA plasmid was 25:1 (2 mg/ml). At this molar ratio, the migration of the peptide–DNA complex was blocked completely (Figure 1).

### Transfection of hepatoma cells with the siRNA-encoding plasmid–CPP complex

**Figure 2:**
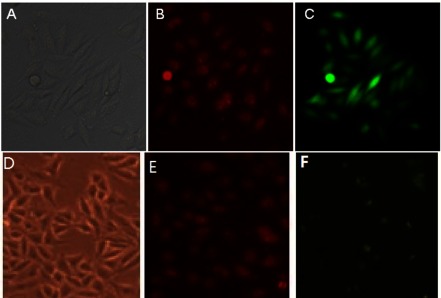
Expression of GFP in transfected hepatoma cells SMMC-7721 cells were transfected with the hTERT-specific siRNA-encoding plasmid–CPP complex (**A**–**C**) or the vector control–CPP complex (**D**–**F**). The CPPs were 5-carboxytetramethylrhodamine-labelled for monitoring the penetrating efficiency. The expression of the plasmid-encoded GFP marker was assessed by monitoring the GFP fluorescence (**C** and **F**) 48 h after transfection under a fluorescence microscope. Phase-contract images of the cells (**A** and **B**) and images of 5-carboxytetramethylrhodamine-labelled CPPs (**B** and **E**) are also shown (magnification, 200×).

SMMC-7721 hepatoma cells were transfected with the plasmid–CPP complex; the transfection efficiency was assessed by monitoring the fluorescence of the plasmid-encoded GFP marker. The fluorescence images showed that the siRNA-encoding plasmid carrying GFP marker was transfected into SMMC-7721cells (Figure 2). Intense GFP-fluorescence emission indicates that 48 h is sufficient to achieve high transfection efficiency (Figure 2C). Based on these data, further analyses of changes in gene expression were conducted on cells collected 48 h after transfection.

### Effect of the plasmid–CPP complex on cell cycle

**Figure 3:**
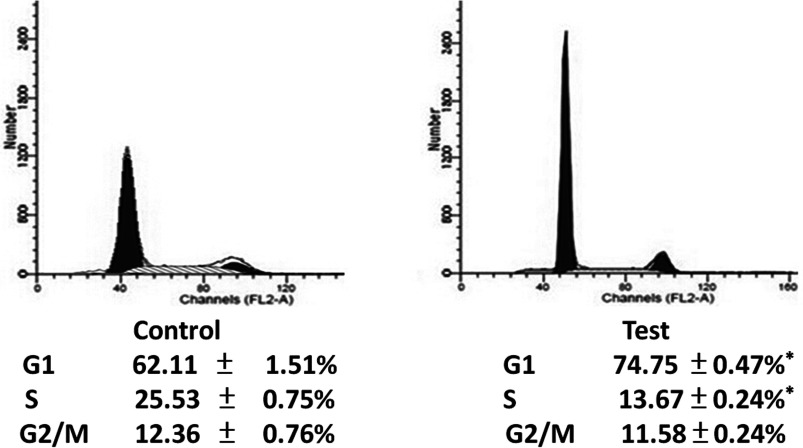
Induction of G_1_-arrest in hepatoma cells transfected with the hTERT-specific siRNA-encoding plasmid–CPP complex SMMC-7721 cells were transfected with the hTERT-specific siRNA-encoding plasmid–CPP complex (test) or the vector control–CPP complex (control). The cell cycle status of the cells was analysed using flow cytometry. Note the decrease in the proportion of cells at G_2_- and S-phases and the increase in the proportion of cells at G_1_-phase, in the Test sample. *P<0.05 compared with control cells.

Flow cytometric analysis was used to evaluate the effect of transfecting the plasmid–CPP complex on cell cycle regulation in SMMC-7721 cells. The data showed that, compared with the cells transfected with the control vector–CPP complex, transfection with the hTERT-specific siRNA-encoding plasmid–CPP complex increased the proportion of cells in the G_1_-phase and decreased those in the G_2_- and S-phase (Figure 3). These data indicate that transfection with the hTERT-specific siRNA plasmid–CPP complex-induced cell-cycle arrest at the G_1_-phase.

### Expression of hTERT mRNA

**Figure 4:**
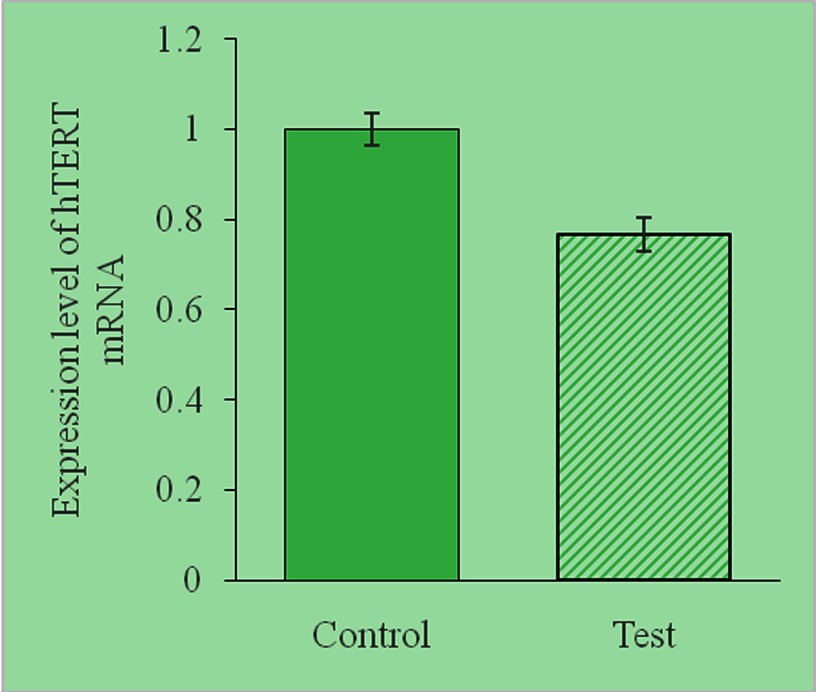
Relative expression of hTERT mRNA in SMMC-7721 cells The expression of hTERT mRNA in SMMC-7721 cells transfected with the hTERT-specific siRNA-encoding plasmid–CPP complex (test) or the vector control–CPP complex (control) was assessed using real-time RT-PCR. The relative mRNA expression levels were calculated using the ΔCt method, with β-actin mRNA as the internal control. Note the suppression of hTERT mRNA expression in the test cells (reduction in relative hTERT expression by 26%; *P*<0.05, as calculated using the Student's *t*test; compared with the control cells).

After transfection with the plasmid–CPP complexes, the level of hTERT mRNA was analysed using real time-PCR. The PCR amplification data from which the Ct values were obtained are shown in Table 2. Treatment of hepatoma cells with the hTERT-specific siRNA plasmid–CPP complex reduced the expression of hTERT mRNA by 26% (Figure 4), indicating suppression of hTERT mRNA expression in hepatoma cells.

**Table 2:** Expression of hTERT mRNA in SMMC-7721 cells

Group	Ct value
hTERT*	
siRNA plasmid-treated cells	13.115±0.327†
Vector control cells	11.83±0.291
**GAPDH**	
siRNA plasmid-treated cells	28.325±0.672
Vector control cells	27.475±0.585

*The relative expression of hTERT mRNA was measured using a real-time RT-PCR assay. The values shown are the mean (*n*=3; each sample was analysed in triplicate and three independent trials of the experiment were performed).

†*P*<0.01, as calculated using the Student's *t*test, compared with control cells.

### hTERT-specific siRNA-mediated inhibition of cell proliferation

The effect of the hTERT-specific siRNA on cell proliferation was evaluated using the MTT assay. The data showed that there was a statistically significant difference in the inhibition of proliferation of SMMC-7721 hepatoma cells between the hTERT-specific siRNA plasmid and the vector control plasmid s (*P*<0.001; Table 3). The inhibition rate in the cells transfected with the hTERT-specific siRNA plasmid was 34.81%, compared with the control cells. The results indicate that the CPP complex with the siRNA-encoding plasmid was delivered into the target cells and that the plasmid-encoded genes (hTERT siRNA and GFP) suppressed the expression of endogenous hTERT, leading to cell-cycle arrest and inhibition of proliferation.

**Table 3:** Inhibition of proliferation of SMMC-7721 cells upon transfection with the hTERT-specific siRNA-encoding plasmid–CPP complex

Group	D*
siRNA plasmid-treated cells	0.1344±0.0051†
Vector control	0.1908±0.0069^‡^

*D, attenuance at 490 nm. Cell proliferation was analysed using the MTT assay.

†The values shown are the mean±S.D. (*n*=3; each sample was analysed in triplicate and three independent trials of the experiment were performed).

‡*P*<0.001, as calculated using the Student's *t*test, compared with control cells.

## DISCUSSION

Our study demonstrated the effectiveness of MMP2 aCPPs as a non-viral method of introducing hTERT siRNA into hepatoma cells to suppress the expression of hTERT. The suppression of hTERT expression was evidenced by the significant decrease in mRNA expression and also by the arrest in cell cycle, a process that depends on hTERT.

As the cancer-associated proteases, MMPs are probably the most-studied with regard to tumour-responsive drug delivery. MMPs are frequently overexpressed in human tumours [24,25]; there is a clear correlation between overexpression of MMPs and poor clinical outcome in many cancers. Therefore, MMPs are attractive targets for protease-sensitive drug delivery. MMP-2, in particular, has been shown to be required for switching the tumour from pre-angiogenic to angiogenic phenotype in chondrosarcomas [10]. In a study of CPP-mediated tumour-targeting, a chemotherapy agent, cisplatin, was complexed with a peptide substrate and incorporated into polethylglycol–hydrogel wafers [26]. The release of the drug from the MMP-activatable wafers into cell culture media was shown to be dependent on MMP-2 and MMP-9. MMP-2 peptide substrates have also been used in delivery of other cargos, including a methotrexate–CPP–dextran conjugate [27,28] and iron oxide nanoparticles [29]. In the present study, we used an aCPP construct that could only be activated if the linker between the CPP and polyanionic peptides is cleaved by MMP2 secreted by tumour cells. Our data showed that the CPP and its cargo (hTERT siRNA) successfully entered hepatoma cells, which could have only happened after cleavage of the linker by MMP2.

In the present study, the delivery of siRNA was mediated by the CPP, which is independent of the siRNA sequence. Therefore, the same CPP carrier can be used or adapted for delivery of other siRNAs. Previous studies have confirmed that the CPP-mediated siRNA delivery systems exert a similar effect in different cell lines [30,31], with little cytotoxicity. It has been shown that the penetration of the CPP–DNA complex did not depend on the endosomal pathway, which would enable a more prolonged effect of the DNA upon intracellular delivery due to a slower release [32–34]. Govindarajan et al. [35] successfully synthesized a CPP, tetramethylammonium fluoride (TMAF), targeting human epidermal growth factor receptor 2 (HER2) and carrying anti-tissue factor (TF) shRNA as cargo in *in vitro* experiments. They found that this CPP–cargo system effectively reduces the volume of tumours xenografted in mice.

CPPs are a promising method for delivering therapeutic agents specifically to tumour cells. They have many advantages, including the ease of preparation of synthetic peptides, use of one peptide for a variety of cargos, obviating the necessity for chemical modification of cargos and increased circulation time of cargo due to protective/shielding effects exerted by the peptide [21,36]. There are still some limitations in our study. Our data showed a moderate suppression of hTERT mRNA expression, which might be due to the low transfection efficiency. Further study should be conducted to optimize the experiment conditions or modify the construct design to improve the transfection efficiency and suppression of target gene. Secondly, we did not assess the hTERT function in the hTERT-specific siRNA-encoding plasmid–CPP complex-treated cells. Thirdly, it will be better to include a control cell which does not express MMP2 to evaluate the aCPPs. These limitations should be addressed in the future study. In addition, we would like to focus on addressing the generalizability of the CPP-mediated plasmid DNA delivery system utilized in the present study, by evaluating its ability to deliver plasmid DNA encoding siRNA targeting other cancer-associated genes into other types of cancer cells.

## CONCLUSIONS

In summary, we designed and tested the efficacy of a tumour-targeted CPP with an MMP2 cleavage site in delivering plasmid DNA encoding hTERT-specific siRNA. Our data suggest that this delivery system can be used to transmit plasmid DNA in cultured hepatoma cells. Further studies are warranted to determine whether this delivery system is generalizable to other cancer cells and other cargos such as plasmid-encoded siRNAs targeting oncogenes. CPPs offer a promising strategy for delivering a variety of cargos to specifically targeted tumour cells and further development of CPP-based cancer therapeutics is necessary to improve their clinical applicability.
